# A Rational Analysis on Key Parameters Ruling Zerovalent Iron-Based Treatment Trains: Towards the Separation of Reductive from Oxidative Phases

**DOI:** 10.3390/nano11112948

**Published:** 2021-11-03

**Authors:** Iván Sciscenko, Antonio Arques, Carlos Escudero-Oñate, Melina Roccamante, Ana Ruiz-Delgado, Sara Miralles-Cuevas, Sixto Malato, Isabel Oller

**Affiliations:** 1 Departamento de Ingeniería Textil y Papelera, Universitat Politècnica de València (UPV), Plaza Ferrándiz y Carbonell s/n, 03801 Alcoy, Spain; ivsci@txp.upv.es (I.S.); aarques@txp.upv.es (A.A.); 2 Institute for Energy Technology (IFE), Instituttveien 18, Kjeller, 2007 Lillestrom, Norway; carlos.escudero@ife.no; 3 CIEMAT-Plataforma Solar de Almería, Carretera de Senés, km 4, 04200 Tabernas, Spain; mroccamante@psa.es (M.R.); anruiz@psa.es (A.R.-D.); smalato@psa.es (S.M.); 4 CIESOL, Joint Centre of the University of Almería-CIEMAT, Ctra. Sacramento, s/n, La Cañada, 04120 Almería, Spain; 5 Programa Institucional de Fomento a la I+D+i, Universidad Tecnológica Metropolitana, Av. Ignacio Valdivieso 2409, San Joaquín, Santiago 8940000, Chile; sara.miralles@psa.es

**Keywords:** Fenton reaction, heterogeneous catalysis, pollutant reactivity enhancement, contaminants of emerging concern, zerovalent iron

## Abstract

The development of treatment trains for pollutant degradation employing zerovalent iron has been attracting a lot of interest in the last few years. This approach consists of pre-treatment only with zerovalent iron, followed by a Fenton oxidation taking advantage of the iron ions released in the first step. In this work, the advantages/disadvantages of this strategy were studied employing commercial zerovalent iron microparticles (mZVI). The effect of the initial amount of mZVI, H_2_O_2_, pH, conductivity, anions and dissolved oxygen were analysed using *p*-nitrobenzoic acid (PNBA) as model pollutant. 83% reduction of PNBA 6 µM into *p*-aminobenzoic acid (PABA) was achieved in natural water at an initial pH 3.0 and 1.4 g/L of mZVI, under aerobic conditions, in 2 h. An evaluation of the convenience of removing mZVI after the reductive phase before the Fenton oxidation was investigated together with mZVI reusability. The Fenton step against the more reactive PABA required 50 mg/L of H_2_O_2_ to achieve more than 96% removal in 15 min at pH 7.5 (final pH from the reductive step). At least one complete reuse cycle (reduction/oxidation) was achieved with the separated mZVI. This approach might be interesting to treat wastewater containing pollutants initially resistant to hydroxyl radicals.

## 1. Introduction

Pesticides, pharmaceuticals, flame retardants and many other chemical substances, belong to the category of so-called contaminants of emerging concern (CECs). These substances are present in urban wastewater in trace concentrations (ng/L–µg/L), and generally they are non-biodegradable, exhibiting low, or incomplete, degradation in conventional wastewater treatment plants technologies. Therefore, these substances are very likely to enter the natural environment and generate ecosystem damage due to plausible side effects on aquatic organisms [1]. In consequence, advanced treatments are required in order to reach the standards of quality for wastewater effluents [2].

In the past three decades, studies applying advanced oxidation processes (AOPs) as tertiary wastewater treatment have demonstrated great effectiveness in the removal of a wide range of CECs [3,4], yet some of them, such as nitroaromatic [5], halogenated [6] or neonicotinoids [7], exhibit slow degradation rates, even with AOPs.

Thorough research has long been performed regarding zerovalent iron (ZVI) applicability in groundwater and wastewater remediation. Its low cost, minor environmental impact and low toxicity had propelled its study towards a wide array of target pollutants. Several works have reported an excellent performance of ZVI in the removal of e.g., heavy metals, nitrates, azo dyes, and halogenated and nitro organic compounds [8,9,10]. On the other hand, the use of iron compounds is also widely spread within the AOPs. Fenton reaction is a very well-known process where the highly reactive HO^●^ are generated in the H_2_O_2_ decomposition mechanism catalysed by Fe^2+^/Fe^3+^, which, in addition, can be enhanced by the action of UV-light (photo-Fenton) [11,12]. Most recently, increasing interest towards the use of ZVI replacing iron salts within the Fenton process has arisen due to its effectiveness for pollutant degradation in mild conditions and its larger versatility. Such an approach exhibits the additional advantage of the easy separation of ZVI after pollutant abatement [13].

Despite the results showing that ZVI-based (or iron oxides-based) Fenton reactions can be more effective at circumneutral pH conditions than homogeneous ones, ZVI technology has proven to be truly successful towards pollutant removals only under acidic conditions. For instance, for reduction reactions, the major restriction is getting rid of the passive oxide layer to continuously expose the ZVI core to the solution [14]. Pre-treatments such as acid-washing or ultrasound have clearly enhanced the process at neutral pH [15]. In the case of Fenton-type processes, iron precipitation significantly drops pollutant oxidation rates. Therefore, the use of iron chelating agents, like ethylenediamine-N,N′-disuccinic acid (EDDS), have also notably improved pollutant abatement rates at circumneutral pH [16]. Evidently, all these solutions arisen to drive ZVI processes at mild conditions notably increase the treatment costs.

An appealing alternative for ZVI-based Fenton processes is splitting them into two individual steps, the first one with ZVI alone, followed by a second (photo)-Fenton step. The main advantages of this strategy could be enhancement of the parent pollutant reactivity by removing the groups that inhibit oxidation steps (i.e., dehalogenation) [17], less H_2_O_2_ consumption and possible use in continuous mode, which can certainly facilitate the scaling up of the process [18]. In this regard, few works investigating this approach have been published. According to our knowledge, the first work coupling ZVI pre-reduction/Fenton processes was performed by Oh and co-workers for the elimination of 2,4,6-trinitrotoluene and hexahydro-1,3,5-trinitro-1,3,5-triazine in ammunition industry wastewater treatment [19]. Similarly, nitrobenzene removal with this treatment train strategy has proven to significantly avoid the production of 1,3-dinitrobenzene (which is 30 times more toxic than the parent compound) that is normally generated in the classical Fenton process [20]. The idea behind these two examples is that after ZVI pre-treatment, the electron-withdrawing nitro group (-NO_2_) was changed into the electron-donor one, amino (-NH_2_), hence, increasing the kinetic rate constant of the original pollutant against HO^●^, which for nitrobenzene represent about 3–4 folds increment [21]. Interesting results can also be found for textile industry wastewater, pre-reducing the azo (-N=N-) group to -NH_2_, thus producing the molecule cleavage, decolorizing the wastewater, as well as increasing the reactivity of the generated by-products shortly before Fenton oxidation [22,23]. Information about nitroaromatic compounds uses, water occurrence, toxicity and remediation difficulties (both, biological and by AOPs) can be found elsewhere [24,25,26].

The feasibility of ZVI reduction/oxidation (by addition of H_2_O_2_) system has been studied towards the degradation of the model pollutant, *p*-nitrobenzoic acid (PNBA), at low concentration levels and applying the innovative two-step strategy: a ZVI pre-treatment (reduction, R 1) followed by the Fenton oxidation reaction by employing commercial ZVI microparticles (mZVI).

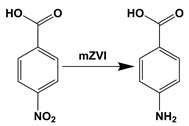
(R 1)

With the main objective of investigating the advantages and disadvantages of increasing the reactivity of the chosen model pollutant in a Fenton-like oxidation process by employing the above-described two-steps treatment train, the effect of dissolved oxygen, pH, water ionic content and the initial amount of mZVI were analysed. Different initial H_2_O_2_ concentrations were studied for the subsequent Fenton process as well as the light irradiation effect. A comparison between separating, or not, the mZVI previous to H_2_O_2_ addition, was also assessed. In addition, consecutive reductions as well as mZVI reusability, were also investigated. Overall, the experiments gathered in this work might be useful to better comprehend the viability of this interesting approach (ZVI for reduction/oxidation combined system), as well as finding the best possible scenarios where efforts on its application should be tackled.

## 2. Materials and Methods

### 2.1. Reagents

Model contaminants, *p*-nitrobenzoic acid (PNBA, C_7_H_5_O_2_NO_2_) and *p*-aminobenzoic acid (PABA, C_7_H_5_O_2_NH_2_) (>99%), and hydrogen peroxide (H_2_O_2_) (35% w/w) (for Fenton reactions) were purchased from Sigma-Aldrich (Burlington, MA, USA). The pH was adjusted employing sodium hydroxide (NaOH) or sulfuric acid (H_2_SO_4_) (96%), both obtained from J. T. Baker (Radnor, PA, USA). Ferrous ion (Fe^2+^) and total iron (Fe_total_) were measured employing acetic acid (CH_3_COOH, J. T. Baker), ammonium acetate (NH_4_CH_3_COO, Riedel-de-Haën, Charlotte, NC, USA), 1,10-phenantroline (C_12_H_8_N_2_, Merck, Darmstadt, Germany) and ascorbic acid (C_6_H_8_O_6_, Sigma-Aldrich). Quantification of H_2_O_2_ was performed with titanium oxysulfate (TiOSO_4_, Fluka, Charlotte, NC, USA). Water matrix effect was evaluated with the addition of sodium sulphate (Na_2_SO_4_, Panreac, Barcelona, Spain), sodium chloride (NaCl, Merck) and sodium bicarbonate (NaHCO_3_, Panreac). High-pressure liquid chromatography (HPLC) measurements were carried out with formic acid (HCOOH) and HPLC-grade acetonitrile (CH_3_CN), both purchased from Panreac. mZVI (>98%) were provided by BASF (Ludwigshafen am Rhein, Germany).

Demineralised water (DW) and natural water (NW) (physicochemical characterization shown in Table 1) were used as water matrices. DW was obtained by the reverse osmosis + electrode ionization plant located at Plataforma Solar de Almería (conductivity < 10 µS/cm and inorganic and dissolved organic carbon below 0.5 mg/L). Respective PNBA and PABA 600 µM stock solutions were prepared in DW in basic media. Both were stable at dark conditions as no hydrolysis was observed.

### 2.2. Chemical Analysis

Total inorganic carbon (TIC) and total organic carbon (TOC) were measured with a TOC-VCN instrument (Shimadzu, Kyoto, Japan). Solution conductivity was determined with a GLP 31 conductivity meter (Crison, Barcelona, Spain). Dissolved oxygen was measured with a LAQUA WQ-310 instrument (Horiba, Kyoto, Japan). H_2_O_2_ was measured spectrophotometrically according to DIN 38402H15 employing an Evolution 220 spectrophotometer (Thermo Fisher Scientific, Waltham, MA, USA). The latter equipment was also employed for Fe^2+^ and Fe_total_ determinations according to ISO 6332:1988. Before total carbon and spectrophotometric analyses, samples were filtered through a 0.45 μm nylon filter (Millipore, Burlington, MA, USA).

Ion chromatography was applied for anions and cations determination (850 Professional IC, Metrohm, Herisau, Switzerland) equipped with Metrohm 872 Extension Module 1 and 2). For cations analyses, a C4-250/4.0 column (5 µm, 4.0 mm × 250 mm Metrosep, Metrohm) was used, with an isocratic flow of the mobile phase, a solution of acetone (9%), nitric acid 2 M (0.085%) and pyridine (0.75%). As for anions′ analysis, a Metrosep A Trap1-100/4.0 column (570 µm, 4.0 mm × 100 mm) was used, and the mobile phase consisted of a NaCO_3_ 5 mM solution injected with a gradient flow.

PNBA and PABA were monitored with an 1100 series HPLC system (Agilent Technologies, Santa Clara, CA, USA) equipped with an UV-DAD detector and a C18 analytical column (Luna 5 µm 100 Å, Phenomenex, Torrance, CA, USA). Nine mL of sample were filtered through 0.2 µm PTFE Millipore filter and mixed with 1 mL CH_3_CN. Analytes were eluted with an isocratic flow of 0.5 mL/min of 80% HCOOH (25 mM) and 20% CH_3_CN, measured with fixed wavelengths of 265 and 275 nm for PNBA and PABA, respectively (detection limit 0.25 μM in both cases).

### 2.3. Experimental Procedure

Pre-reductive reactions were carried out in an open batch reactor containing 1 L of PNBA as the target pollutant. Two initial concentrations of PNBA were used, 6 and 60 µM, thus simulating two possible scenarios; the lowest simulating the concentrations found in urban wastewater effluents, and the highest simulating highly polluted waters. pH was adjusted to 3.0, 5.0 or 7.0 with H_2_SO_4_ or NaOH 1 M when needed, and mZVI (0.056, 0.56, 1.4, 2.8 and 4.2 g/L) was added directly afterwards under continuous stirring. To study the effect of O_2_, the same procedure as above was followed but with previous N_2_ purging (5 min of N_2_ bubbling before adding mZVI). Regarding the anions effect study, SO_4_^2−^, Cl^−^ and HCO_3_^−^ salts were added to DW until obtaining similar conductivity as NW, requiring 400 mg/L of Na_2_SO_4_, 350 mg/L of NaCl and 700 mg/L of NaHCO_3_. Afterwards, H_2_SO_4_ 1 M was added to reach pH 3.0 and then 1.4 g/L of mZVI were added to the solution.

**Figure 1:**
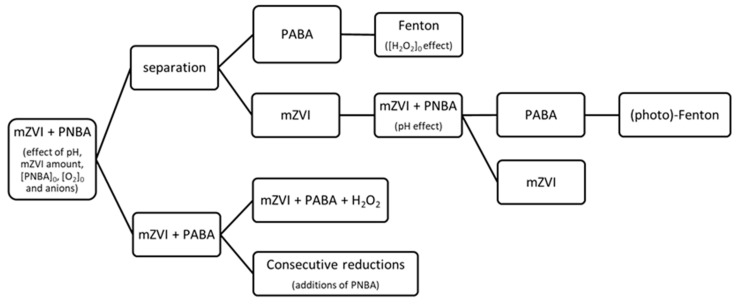
Summary of the studied processes in this work.

Fenton reactions were performed in two different ways: in one case by adding 50 mg/L of H_2_O_2_ directly inside the reactor containing the mZVI, and in the other one, by first waiting for the settling of mZVI and transferring the supernatant (800 mL) to an analogous reactor previous addition of H_2_O_2_ (10, 25 and 50 mg/L). In the second case, mZVI reusability (pH_0_ 3.0 and 7.0) was carried out with the 200 mL remaining in the bottom of the reactor with the mZVI that could not be separated and topping up with 800 mL of PNBA. As an individual case, for the mZVI reuse at pH 7.0, photo-Fenton (carried out in a Atlas Suntest XLS+ solar simulator, Mount Prospect, IL, USA) was also tested. The experiments were carried out at 30 W/m^2^ of UVA irradiance. The solar simulator offers an emission range of 250–765 W/m^2^ (complete spectral emission, 300–800 nm). The system was always agitated with a magnetic stirrer. The experimental procedure followed is represented in the scheme of Figure 1. In all cases, the experiments started with pH = 3.0, mZVI = 1.4 g/L and PNBA 60 μM, while the initial concentration of PABA in Fenton and photo-Fenton assays was the one obtained after the reduction step.

## 3. Results and Discussion

### 3.1. Pre-Reductive Step

#### 3.1.1. Concentration of Micro ZVI and pH Effect

**Figure 2:**
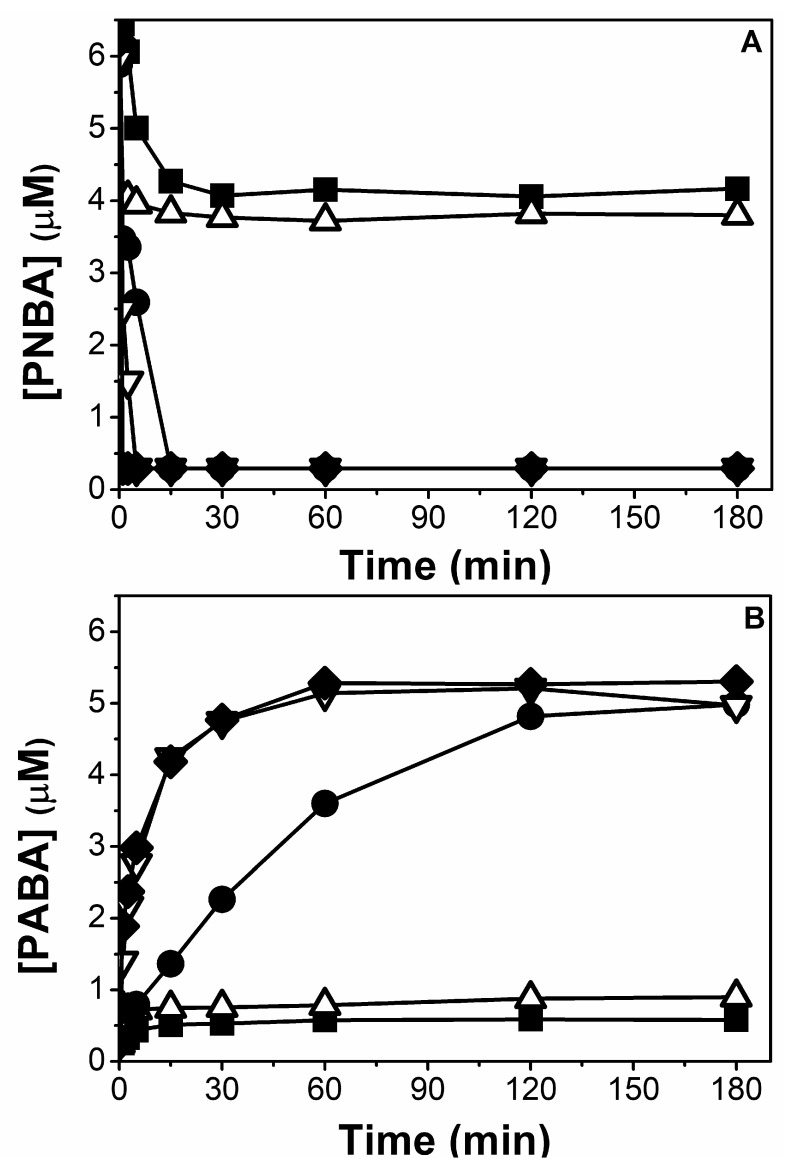
PNBA reduction (**A**) and generated PABA (**B**) in NW at pH 3.0 with different amounts of mZVI (g/L): 0.056 (■), 0.56 (△), 1.4 (●), 2.8 (▽) and 4.2 (◆).

Firstly, the effect of the initial amount of mZVI was explored in the range 0.056–4.2 g/L at pH 3.0 in NW (Figure 2). Results indicated that only when mZVI was ≥1.4 g/L, 6 μM of PNBA was efficiently reduced. However, the further addition of mZVI did not result in a significant enhancement of the reductive process. This issue could be attributed to the parallel reactions of mZVI with water (R 2 and R 3), which compete with the redox process involving the pollutant (R 1); only when there is enough mZVI availability, pollutant reduction occurs. As predicted by reactions R 2 and R 3, a fast pH increment was observed, reaching its final value in the first 5 min and remaining constant after up to 180 min. When using 0.056 and 0.56 g/L of mZVI, pH reached 5.5 in both cases, whereas from 1.4 to 4.2 g/L, it attained 7.5 to 8.0.

Fe^0^ + 2H^+^ → Fe^2+^ + H_2_↑ (R 2)

2Fe^0^ + 2H_2_O + O_2_ → 2Fe^2+^ + 4OH^−^ (R 3)

4Fe^2+^ + 4H^+^ + O_2_ → 4Fe^3+^ + 2H_2_O (R 4)

Fe^0^ + 2H^+^ + O_2_ → Fe^2+^ + H_2_O_2_ (R 5)

Dissolved Fe_total_ concentration levels at the end of the assay were considerably high, 27 mg/L at 180 min when starting with the minimum mZVI quantity of 0.056 g/L, 30 mg/L for 0.56 g/L of mZVI, 40 mg/L for 1.4 g/L of mZVI, 43 mg/L for 2.8 g/L of mZVI and 45 mg/L with 4.2 g/L of mZVI. Moreover, in all cases, no significant differences were observed between Fe^2+^ and aforementioned Fe_total_ concentrations, indicating negligible Fe^3+^ presence.

Since mZVI consumes O_2_ (e.g., reactions R 3 to R 5) and the likelihood formed Fe^3+^ rapidly precipitates as (oxy)hydroxides, due to the high amount of mZVI, anaerobic conditions can be rapidly reached (>99% of O_2_ consumption in 2 min, remaining constant until the end of the assay, see Figure S1), thus explaining the high Fe^2+^ concentration values leached at neutral pH [27].

PNBA (6 µM) reduction was also studied at pH 5.0 and 7.0 with 1.4 g/L of mZVI in NW. In sharp contrast, incomplete reductions were obtained, attaining 23% PNBA removal in 180 min at pH 5.0 and negligible at pH 7.0 (data not shown). This can be explained by the enhanced iron corrosion that occurs in acidic vs. neutral media, which in turns favours pollutant reduction [28].

**Figure 3:**
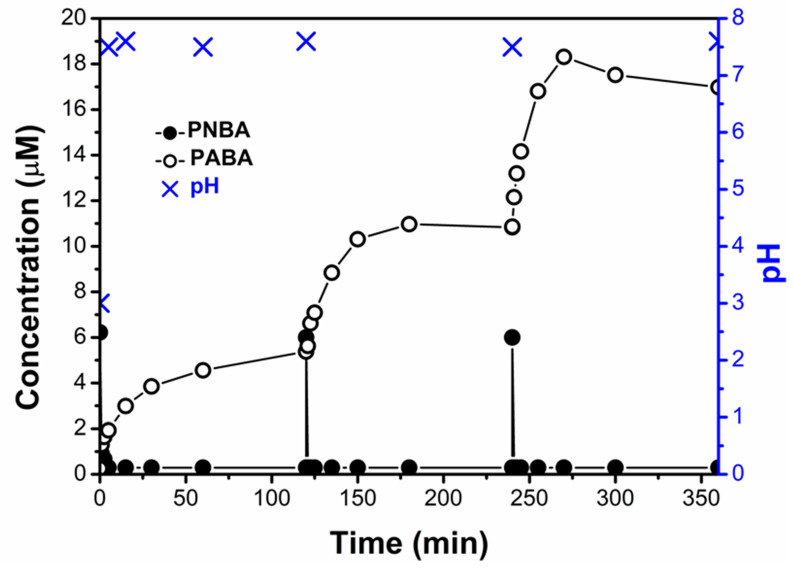
Three consecutive reductions of PNBA 6 µM (additions each 120 min) in NW with 1.4 g/L of mZVI and initial pH 3.0.

**Figure 4:**
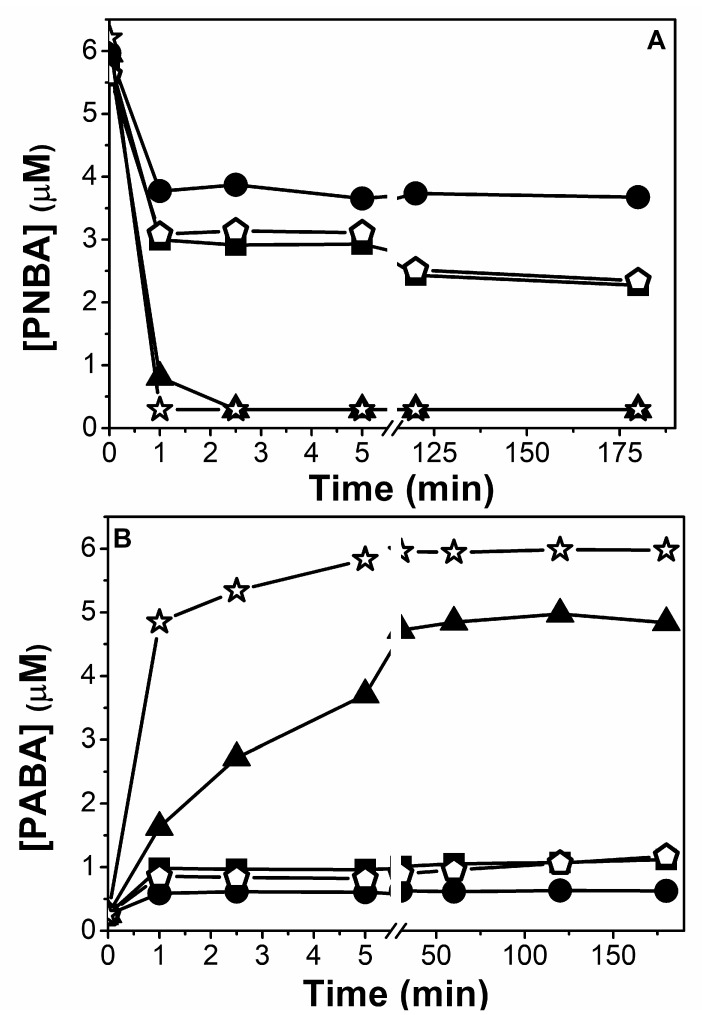
Water matrix effect. PNBA 6 µM reduction with mZVI 1.4 g/L at initial pH 3.0 in DW, (**A**) PNBA and (**B**) PABA kinetics, respectively. Symbols are represented by: DW alone (⬠), DW + Cl^−^ 212 mg/L (●), DW + SO_4_^2−^ 270 mg/L (■), DW + TIC 34 mg/L (▲), and DW + N_2_ purging (☆).

The experiment was repeated (pH = 3.0, 1.4 mg/L of mZVI, NW) but adding three consecutive doses of PNBA 6 µM. Interestingly, complete PNBA reduction was observed in each cycle, despite the fact the initial pH was not corrected, and hence, it was systematically above 7 (Figure 3). Different reasons must be considered simultaneously to explain this behaviour: (i) activation of mZVI with the first treatment at pH 3.0 removing the iron oxides passive layer, thus having more exposed Fe^0^ [29], (ii) the O_2_ was rapidly consumed (Figure S1), enhancing the reductive process (see later Figure 4), and (iii) the likelihood generation of magnetite (Fe_3_O_4_) and/or Fe^3+^ (oxy)hydroxides with adsorbed Fe^2+^, which are also well known to easily reduce nitroaromatic compounds at neutral pH [30,31,32].

Finally, it is important to indicate that the initial concentration of PNBA did not play an important role in the studied range, as comparable results were obtained when selected reactions were performed with 60 µM of PNBA (pH = 3, ZVI = 1.4 g/L and NW as matrix).

#### 3.1.2. Water Matrix and O_2_ Effect

When DW was employed (PNBA 6 μM, pH 3.0, 1.4 g/L mZVI) an incomplete removal was obtained, reaching a fast 50% of PNBA elimination in ca. 2.5 min, but followed by a very slow decay up to 2.3 µM in 180 min (Figure 4A). Therefore, a NW matrix favoures the reductive process.

Water ionic constitution effects have long been studied for different target pollutants as they involve many complex reactions, explaining the discrepancy reported for pollutant removal with ZVI. For instance, As(V) reduction rates decrease in the presence of different anions, being this inhibition effect more important when HCO_3_^−^ is present [33]. Similarly, trichloroethylene dehalogenation by ZVI nanoparticles was significantly reduced by the presence of anions [34]. On the other hand, Yin and co-workers observed that Cl^−^ continuously favoured nitrobenzene reduction when gradually increasing its concentration from 0 to 1 g/L, whereas this only happened in a certain concentration range for SO_4_^2−^ and HCO_3_^−^ [35].

Therefore, to investigate which anion might be the main responsible for improving PNBA reduction in NW, the individual effect of SO_4_^2−^, Cl^−^ and HCO_3_^−^ was studied, all of them added into DW as sodium salts until reaching conductivity of 750 µS/cm, which was that of NW (Table 1).

As shown in Figure 4, no significant differences in reduction of PNBA, or generation of PABA, were observed between DW with and without 270 mg/L of SO_4_^2−^. With 212 mg/L of Cl^−^, a slight inhibition was observed (PNBA 3.5 μM and PABA 0.6 μM final concentrations). Only when employing 508 mg/L of HCO_3_^−^ (remaining a TIC content of 34 mg/L after pH adjustment to 3.0, shortly before mZVI addition), rapid PNBA reduction occurred, achieving the detection limit (>96% of PNBA reduction) in ca. 2.5 min and obtaining ca. 2.5 μM PABA formation in the same period, which was 12 times faster than the one obtained in NW (2.5 μM of PABA in 30 min, Figure 2B).

These results are in agreement with the iron corrosion obtained, being at the end of each experiment (180 min) the lowest dissolved Fe_total_ value of 22 mg/L with NaCl, and the highest one, 80 mg/L, when adding NaHCO_3_ (Figure S2A), also in agreement with other previously reported works [36,37]. Besides, since the initial pH was 3.0, it is also known that dissolved CO_2_ can also accelerate ZVI corrosion [37,38]. In line with these statements, TIC of NW at pH 3.0 was 10 mg/L, hence, having intermediate iron corrosion (Fe_total_ = 40 mg/L), and thus also an intermediate PNBA reduction rate, between DW and DW + HCO_3_^−^.

Regarding the effect of O_2_, an almost instant and complete reduction of PNBA in DW was obtained when removing it by purging the reaction mixture with N_2_. This reaction rate could not even be achieved, as stated above, with aerobic NW, where the final PABA concentration was 5 µM instead of 6 µM (Figure 2B). This mass balance defect was explained when performing a blank experiment with PABA 6 μM in presence of 1.4 g/L mZVI in NW under aerobic and anaerobic conditions. PABA concentration in anoxic NW remained constant for 180 min, whereas it decayed 1 µM under aerobic conditions.

It is important to highlight that under anaerobic conditions and at initial acidic pH, reaction R 2 is mainly governing the mZVI/H_2_O system, whereas when O_2_ is present, reactions R 3 and R 4 gain relevance. In this last case, the generated Fe^3+^ eventually leads to rapid iron (oxy)hydroxide formation, and species such as hematite (Fe_2_O_3_), goethite (FeOOH) and ferric hydroxide (Fe(OH)_3_) are produced [11]. Consequently, PABA could be adsorbed as well as the mZVI surface passivated, thus stopping the PNBA reduction. These findings are also in agreement with the observed pH values achieved by the system. In a DW-aerobic system, the pH at the end of the process was 5.5, whereas for DW-anaerobic was 8.0 (Figure S2B), which could be attributed to the aforementioned negligible Fe^3+^ hydrolysis contribution for the last case.

On the other hand, it is reported that in-situ Fenton oxidation might also happen under aerobic conditions in a ZVI/H_2_O system (reactions R 5 to R 8) [15,39], which could be degrading in some extent the formed PABA. In fact, non-identified peaks were observed within the chromatograms (when monitoring PNBA by HPLC) only during the aerobic reduction (Figure S3). In order to evaluate if the Fenton reaction was taking place in the reductive stage, a test with PNBA 6 µM in aerobic DW containing CH_3_CN 10 mM (as HO^●^ quencher) was done, not exhibiting any significant differences with the one without it.

Therefore, it was concluded that PABA oxidation by in-situ Fenton reaction was insignificant compared to its removal by adsorption. Noteworthily, the unknown generated by-products should be, most likely, reduction intermediates containing nitroso or hydroxylamine groups [40,41], just formed and consumed faster under anoxic conditions.

Fe^0^ + H_2_O_2_ → Fe^2+^ + 2OH^−^ (R 6)

Fe^2+^ + H_2_O_2_ → Fe^3+^ + HO^●^ + OH^−^ (R 7)

Fe^3+^ + H_2_O_2_ → Fe^2+^ + HO_2_^●^ + H^+^ (R 8)

### 3.2. Fenton Oxidation Step

As indicated in the Introduction, ZVI can also be used for pollutants oxidation in the presence of hydrogen peroxide, via Fenton or related processes. Two possible procedures were investigated: (i) adding the H_2_O_2_ in the reactor after separating the mZVI (only leached iron remained, therefore only the homogeneous Fenton reaction is taking place) once the reductive stage is finished, and (ii) maintaining mZVI together with H_2_O_2_, so homogeneous (with remaining iron leached in solution) and potential heterogeneous Fenton oxidation may occur. These approaches were tested with PNBA 60 μM, pH = 3.0 and mZVI = 1.4 g/L.

**Figure 5:**
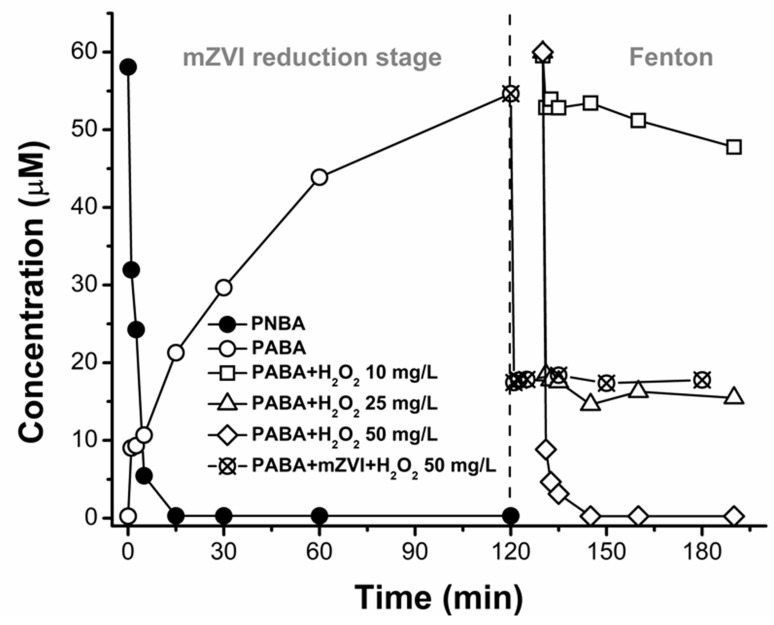
Reduction of PNBA 60 μM in NW at initial pH 3.0 with 1.4 g/L of mZVI, followed by the different tested Fenton oxidations. When needed, 10 min for mZVI separation took place.

Regarding the first strategy, after mZVI decantation and supernatant separation (10 min), the solution exhibited a pH of 7.3, dissolved Fe_total_ of 55 mg/L, and ca. 100 mg/L of non-dissolved (without filtration) Fe_total_. Three different H_2_O_2_ initial concentrations were studied: 10, 25 and 50 mg/L, obtaining 15%, 71% and more than 96% PABA removal in the first 15 min, respectively (Figure 5).

With the second strategy, when performing the Fenton transformation with 50 mg/L of H_2_O_2_ without mZVI previous separation, incomplete removal of PABA was achieved (60% in the first minute, remaining constant afterwards). Moreover, a much faster H_2_O_2_ consumption was also observed. Whereas for the separation strategy a 52% of initial H_2_O_2_ 50 mg/L was consumed in 1 min, in presence of the mZVI was >99% (Figure S4), justified mainly by reaction R 6.

Therefore, by means of H_2_O_2_ consumption and PABA degradation percentage, the most efficient overall PNBA removal was obtained with a first pre-reductive step, the subsequent mZVI separation, and the Fenton homogeneous oxidation at the end. In any case, the reformation of PNBA when performing the Fenton reaction was detected.

Finally, to prove that the pre-reduction step actually enhanced PNBA reactivity, the oxidative Fenton process (with previous mZVI separation system) was also carried out with an additional 60 μM of PNBA (i.e., the supernatant with the formed PABA ca. 60 μM from the previous reduction, and the intentionally added PNBA 60 μM). The data presented in Figure S5 confirms the aforementioned hypothesis, being the PNBA degradation rate almost three times slower than the one of PABA. These results are in agreement with the published HO^●^ kinetic rate constants (k_PNBA-HO_● 2.6 × 10^9^ M^−1^ s^−1^ and k_PABA-HO_● 8.2 × 10^9^ M^−1^ s^−1^) [21].

### 3.3. mZVI Reusability

**Figure 6:**
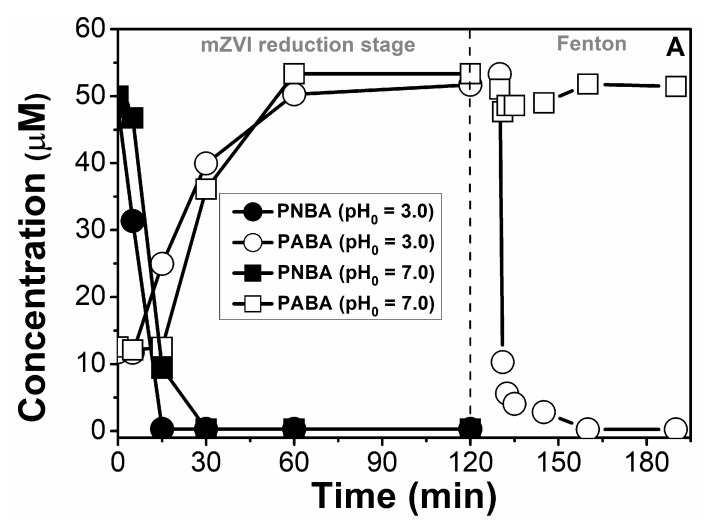

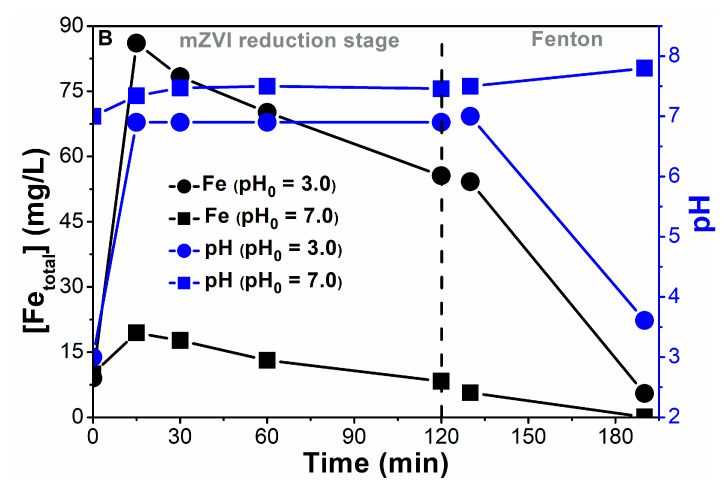
mZVI reuse for new PNBA 60 µM solutions prepared in NW with initial pH 3.0 and 7.0. 10 min for mZVI separation previous H_2_O_2_ 50 mg/L addition in the subsequent Fenton step: (**A**) PNBA decay and PABA formation; (**B**) total dissolved iron and pH are also shown.

As an additional advantage of mZVI separation after the pre-reduction step, its plausible reusability may be mentioned. Therefore, after one complete PNBA 60 μM reduction and mZVI separation from the solution, its reusability was investigated with a new PNBA 60 μM solution in NW. This assay was performed not only at initial pH 3.0 but also at an initial pH of 7.0. Interestingly, a second complete reduction was successfully achieved in 60 min for both cases, regardless of the initial pH (Figure 6A). These results are in line with previously discussed successive reductions (Figure 3), indicating that mZVI requires only a first acidic treatment to be efficient at neutral pH.

When continuing with the subsequent Fenton oxidation, only with the reuse after the first pre-reduction at pH 3.0, fast and complete PABA removal was obtained (almost total in 30 min), whereas with the reuse after pre-reduction at pH 7.0, negligible PABA degradation was observed. This issue was due to the iron lixiviation occurring within the first step. Only when the reductive phase was carried out under acidic conditions, dissolved leached iron was high enough (Fe_total_ = 55 mg/L, Figure 6B) to make the subsequent Fenton oxidation efficient. On the contrary, when starting the pre-reduction step at pH 7.0, dissolved leached iron was only 5 mg/L, hence, not observing any PABA degradation.

**Figure 7:**
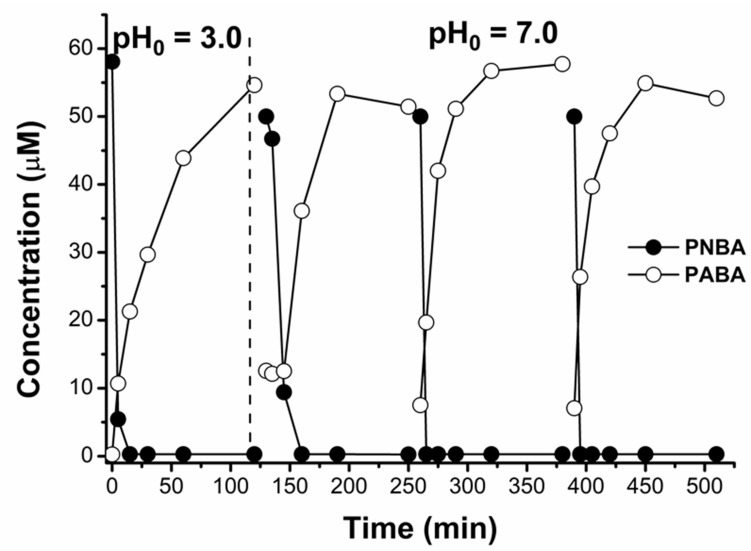
First PNBA 60 μM reduction at pH 3.0 with 1.4 g/L of mZVI in NW, and its subsequent reuses at pH 7.0. 10 min between each cycle was required for mZVI decantation and supernatant separation.

Interestingly, mZVI reusability at pH 7.0 could maintain PNBA reduction, without making the PABA formation rate decrease. Figure 7 shows three mZVI successful reuse cycles after the first activation at pH 3 and without requiring further pH modifications. As previously discussed in Section 3.1.1, this should also be attributed not only to the passive layer removal, but also to the formation of Fe^2+^-Fe^3+^ oxides which can also reduce nitroaromatic compounds at neutral pH [32].

Finally, due to the negligible PABA Fenton oxidation with reused mZVI at pH 7.0 with PNBA 60 μM, the same experiment was fully replicated with an initial PNBA concentration of 6 μM. PABA degradation was now slightly higher than with the system of 60 μM (15% in 120 min). Moreover, although the dissolved Fe_total_ was low, the colloidal iron was still significant (43 mg/L of Fe_total_), so the photo-Fenton process employing simulated sunlight in a solar simulator was also investigated. Nevertheless, no significant differences compared to dark conditions were observed (results not shown).

## 4. Conclusions

A combined reductive/oxidative sequential treatment with commercial mZVI was tested in the removal of PNBA from water under different operating conditions. pH 3.0, high bicarbonate concentrations and the absence of O_2_ notably improved the PNBA pre-reduction step and PABA formation. Besides, the subsequent Fenton oxidation step showed a better performance when mZVI was previously separated for attaining complete PABA oxidation, with also markedly lowered H_2_O_2_ consumption.

Noteworthily, several drawbacks were also observed. Due to parallel reactions with H_2_O and O_2_, a minimum amount of 1.4 g/L mZVI was required to obtain complete PNBA reductions, which is very high considering CEC concentrations (ng–μg/L) normally present in effluents of municipal wastewater treatment plants. Secondly, pH 3.0 was needed to initially reduce PNBA and so to obtain a high residual amount of dissolved iron to ensure an efficient Fenton oxidation within the second step—which, at the same time, generated iron sludge. In this regard, even though PNBA reduction at neutral pH was possible, low iron corrosion was achieved, and therefore, the subsequent Fenton oxidation was not possible anymore.

Possible solutions to the aforementioned drawbacks could be purging N_2_ along the pre-reduction step to reduce the required amount of mZVI, and then reacidification, or adding an iron-chelating agent, for improving the oxidative (photo)-Fenton step. Proposed modifications to the combined reduction/oxidation mZVI system must be analyzed from a life cycle assessment (LCA) perspective to decide their potential and possibly interesting applications.

In any case, this strategy might be extended to other families of compounds that can be reduced, but are difficult to oxidize (nitroaromatic, dyes or halogenated). After reduction, ZVI should be removed in order to avoid undesirable H_2_O_2_ consumption by this metal, what might involve strategies to immobilize iron. Finally, upon irradiation, photo-Fenton occurs oxidizing the organics that are found in the sample.

In conclusion, this approach might be limited to few scenarios, such as highly polluted wastewater containing outstanding levels (mg–g/L) of reducible contaminants, as well as acidic pH, which could be the case of some industries, such as, textile or explosive manufacturers. It can be a plausible alternative for this niche application, replacing the ZVI by Fe(II)-Fe(III) oxides couple system, without separating the catalyst previous to H_2_O_2_ addition, hence, being easier to produce reduction at neutral pH as well as avoiding iron sludge issues in the Fenton step.

## Figures and Tables

**Table 1:** NW characterization.

Parameter	Value
pH	7.6 ± 0.2
Conductivity (µS/cm)	748 ± 4
Dissolved organic carbon (mg/L)	<0.5
Total inorganic carbon (mg/L)	46 ± 4
SO_4_^2−^ (mg/L)	40 ± 10
Cl^−^ (mg/L)	70 ± 20
PO_4_^3−^ (mg/L)	<0.5
ClO^−^ (mg/L)	<0.02
Ca^2+^ (mg/L)	12 ± 3
Mg^2+^ (mg/L)	14 ± 2
K^+^ (mg/L)	2.07 ± 0.08
Na^+^ (mg/L)	110 ± 20

## Data Availability

Data is contained within the article.

## Supplementary Materials

The following are available online at https://www.mdpi.com/article/10.3390/nano11112948/s1, Figure S1: Dissolved oxygen concentration during PNBA 6 μM reduction in NW with 1.4 g/L of mZVI at initial pH 3.0; Figure S2: PNBA 6 µM reduction at initial pH 3.0 with 1.4 g/L mZVI in different water matrices at 180 min: (A) released Fe (measured as total and filtered) (B) pH; Figure S3: Kinetic formation of two unknown compounds, called X1 and X2, observed in the chromatograms during PNBA 6 µM reduction with 1.4 g/L mZVI at initial pH 3.0 in DW under aerobic and anaerobic conditions; Figure S4: H_2_O_2_ consumption during Fenton oxidation experiments, separating the mZVI previous different H_2_O_2_ concentrations, 10 mg/L, 25 mg/L and 50 mg/L, as well as the one containing the mZVI 1.4 g/L with H_2_O_2_ 50 mg/L; Figure S5: Fenton oxidation of the supernatant ([PABA] = 50 μM, [Fe_total_] = 55 mg/L, pH = 7.3) with 50 mg/L of H_2_O_2_, and 50 μM of PNBA (added). Theorical PNBA degradation rate has been also inserted in the graph.
