# Supplementary material for: A Rational Analysis on Key Parameters Ruling Zerovalent Iron-Based Treatment Trains: Towards the Separation of Reductive from Oxidative Phases
Source: Nanomaterials (Basel). 2021 Nov 3;11(11):2948. doi: 10.3390/nano11112948 (PMC8623565; doi:10.3390/nano11112948)
Supplement: Supplementary file 1 [file nanomaterials-11-02948-s001.zip › nanomaterials-1428843-supplementary.pdf]

Supplementary Material

A Rational Analysis on Key Parameters Ruling Zerovalent Iron-Based Treatment Trains: Towards the Separation of Reductive from Oxidative Phases

Iván Sciscenko [1], Antonio Arques [1], Carlos Escudero-Oñate [2], Melina Roccamante [3], Ana Ruiz-Delgado [3,4], Sara Miralles-Cuevas [5], Sixto Malato [3,4] and Isabel Oller [3,4,*]

[1] Departamento de Ingeniería Textil y Papelera, Universitat Politècnica de València (UPV), Plaza Ferrándiz y Carbonell s/n, 03801 Alcoy, Spain; ivsci@txp.upv.es (I.S.); aarques@txp.upv.es (A.A.)
[2] Institute for Energy Technology (IFE), Instituttveien 18, 2007, Kjeller, 2007, Lillestrom, Norway; carlos.escudero@ife.no
[3] CIEMAT-Plataforma Solar de Almería, Carretera de Senés, km 4, 04200, Tabernas, Spain; mroccamante@psa.es (M.R.); anruiz@psa.es (A.R.-D.); smalato@psa.es (S.M.)
[4] CIESOL, Joint Centre of the University of Almería-CIEMAT, Ctra. Sacramento, s/n, La Cañada, 04120 Almería, Spain
[5] Programa Institucional de Fomento a la I+D+i, Universidad Tecnológica Metropolitana, Av. Ignacio Valdivieso 2409, 8940000, San Joaquín, Santiago, Chile; sara.miralles@psa.es
* Correspondence: ioller@psa.es

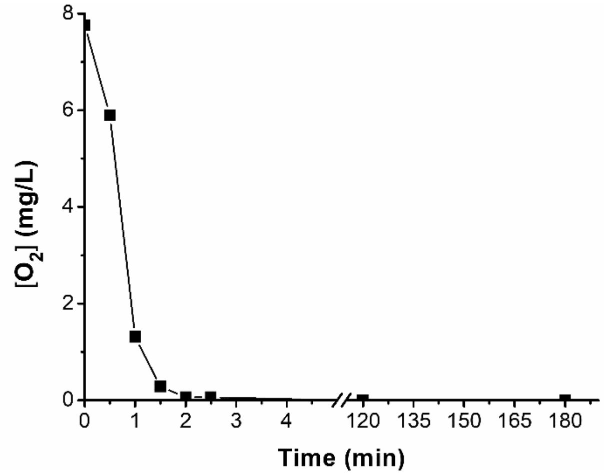

Figure S1. Dissolved oxygen concentration during PNBA 6 μM reduction in NW with 1.4 g/L of mZVI at initial pH 3.0.

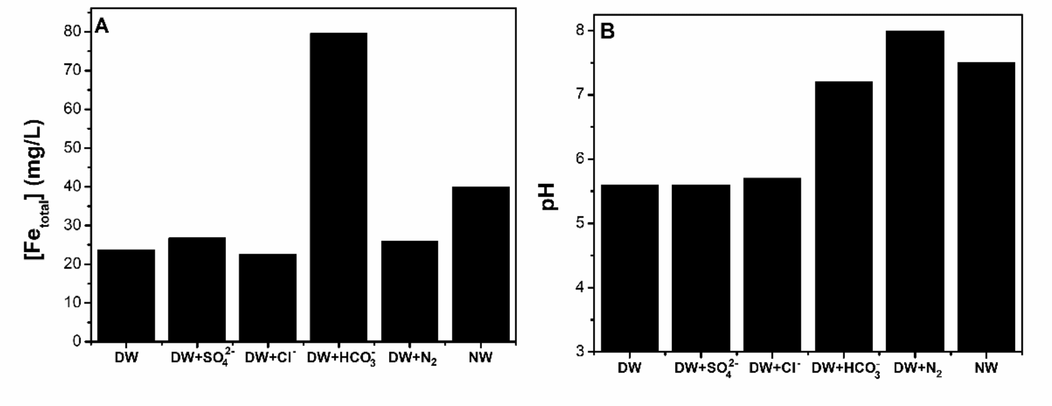

Figure S2. PNBA 6 μM reduction at initial pH 3.0 with 1.4 g/L mZVI in different water matrices at 180 min: A) released Fe (measured as total and filtered) B) pH.

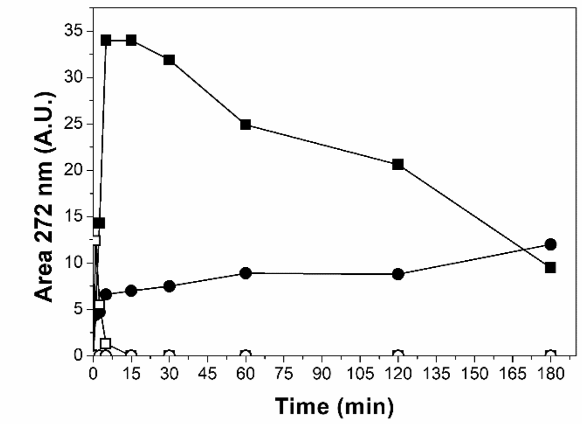

Figure S3. Kinetic formation of two unknown compounds, called X1 and X2, observed in the chromatograms during PNBA 6 μM reduction with 1.4 g/L mZVI at initial pH 3.0 in DW under aerobic (■ and ● for X1 and X2, respectively) and anaerobic (□ and ○ for X1 and X2, respectively) conditions.

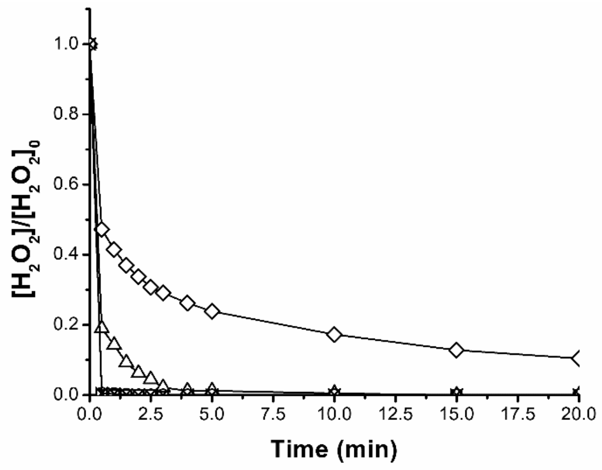

Figure S4. H_2O_2 consumption during Fenton oxidation experiments, separating the mZVI previous different H_2O_2 concentrations, 10 mg/L (○), 25 mg/L (△) and 50 mg/L (◇), as well as the one containing the mZVI 1.4 g/L with H_2O_2 50 mg/L (⊕).

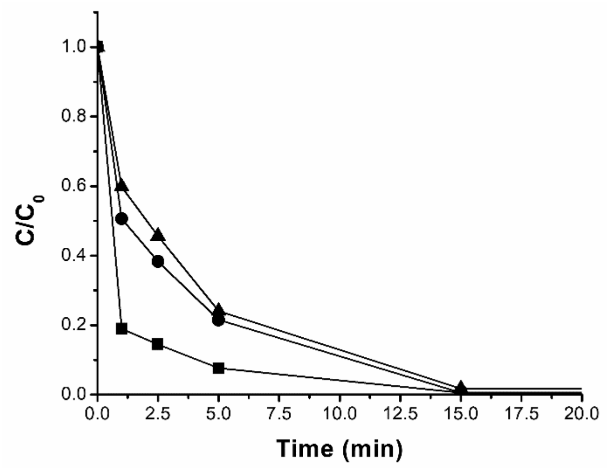

Figure S5. Fenton oxidation of the supernatant ([PABA] = 50 μM, [Fe_{total}] = 55 mg/L, pH = 7.3) with 50 mg/L of H_2O_2, and 50 μM of PNBA (added). Theorical PNBA degradation rate[1] has been also inserted in the graph. Symbol representation has been given by: ■ (PABA), ● (PNBA) and ▲ (theorical PNBA degradation rate).

References

1. Buxton, G.V.; Greenstock, C.L.; Helman, W.P.; Ross, A.B. Critical Review of rate constants for reactions of hydrated electrons, hydrogen atoms and hydroxyl radicals (·OH/·O−) in Aqueous Solution. *J. Phys. Chem. Ref. Data* **1988**, *17*, 513–886, doi:10.1063/1.555805.
